# QuikLitE, a Framework for Quick Literacy Evaluation in Medicine: Development and Validation

**DOI:** 10.2196/12525

**Published:** 2019-02-22

**Authors:** Jiaping Zheng, Hong Yu

**Affiliations:** 1 College of Information and Computer Sciences University of Massachusetts Amherst Amherst, MA United States; 2 Department of Computer Science University of Massachusetts Lowell Lowell, MA United States; 3 Center for Healthcare Organization and Implementation Research Bedford Veterans Affairs Medical Center Bedford, MA United States

**Keywords:** health literacy, psychometrics, crowdsourcing

## Abstract

**Background:**

A plethora of health literacy instruments was developed over the decades. They usually start with experts curating passages of text or word lists, followed by psychometric validation and revision based on test results obtained from a sample population. This process is costly and it is difficult to customize for new usage scenarios.

**Objective:**

This study aimed to develop and evaluate a framework for dynamically creating test instruments that can provide a focused assessment of patients’ health literacy.

**Methods:**

A health literacy framework and scoring method were extended from the vocabulary knowledge test to accommodate a wide range of item difficulties and various degrees of uncertainty in the participant’s answer. Web-based tests from Amazon Mechanical Turk users were used to assess reliability and validity.

**Results:**

Parallel forms of our tests showed high reliability (correlation=.78; 95% CI 0.69-0.85). Validity measured as correlation with an electronic health record comprehension instrument was higher (.47-.61 among 3 groups) than 2 existing tools (Short Assessment of Health Literacy-English, .38-.43; Short Test of Functional Health Literacy in Adults, .34-.46). Our framework is able to distinguish higher literacy levels that are often not measured by other instruments. It is also flexible, allowing customizations to the test the designer’s focus on a particular interest in a subject matter or domain. The framework is among the fastest health literacy instrument to administer.

**Conclusions:**

We proposed a valid and highly reliable framework to dynamically create health literacy instruments, alleviating the need to repeat a time-consuming process when a new use scenario arises. This framework can be customized to a specific need on demand and can measure skills beyond the basic level.

## Introduction

### Background

The past few decades have seen a proliferation of health literacy instruments. Recent reviews have identified dozens of tools [1-5], ranging from general measurements to disease-, content-, or population-specific ones. These instruments aim to measure a variety of skills necessary to function in the health care system. For example, 1 study [5] categorized 51 instruments based on 11 dimensions, including the ability to perform basic reading tasks, to communicate on health matters, and to derive meaning from sources of information. The ability to understand information is 1 of the 4 skills of health literacy identified in a systematic review [6]. It is also one of the most measured skills in the instruments. Those that measure this skill are widely used in research.

Designing an instrument measuring reading ability, or print literacy, is a time- and effort-intensive process. It usually starts with experts curating passages of text or word lists, followed by psychometric validation and revision based on test results obtained from a sample population. Once validated, the instruments stay static.

There are a few potential drawbacks of reusing instruments designed long in the past. First, language use patterns evolve over time. Health literacy, reading ability in particular, needs to adapt to these changes. Instruments that were designed from early text sources may be out of date when employed decades later. Although we are not aware of reports of this nature in the health literacy literature, researchers working on general vocabulary estimation tools have seen the need to update old tests [7].

Moreover, the public’s reading abilities may also change because of increased exposure to print material. Statistics of educational attainment show that the population is receiving more education. Degrees conferred at various postsecondary levels all rose more than 30% over the decade between 2004-05 and 2014-15 according to a recent US national report [8]. More exposure to advanced text material at or above college level may improve one’s reading ability. Older instruments that tend to use low-grade-level text may struggle to distinguish readers proficient above the very basic level that is required to function in the health care system. This ceiling effect, many test takers obtaining perfect scores [9], can be more pronounced when such tests are administered to groups in the general population, reflecting that many were developed with convenience samples of patients in a health care setting. Therefore, they function well as screening tools to detect low health literacy but may fail to properly separate advanced readers.

In this work, we aimed to develop a test framework that can be customized to a specific need on demand and can measure skills beyond the basic level.

### Prior Work

We highlight a few instruments in this section that measure the individual skills and abilities of understanding written text. For a more complete review of instruments that measure both reading and other skills, we refer the reader to a recent review [5].

Numerous instruments have been developed to test health literacy since the 1990s. There are 2 such frequently used instruments: the Rapid Estimate of Adult Literacy in Medicine (REALM) [10] and the Test of Functional Health Literacy in Adults (TOFHLA) [11], with its shortened form Short Test of Functional Health Literacy in Adults (S-TOFHLA) [12].

REALM is a tool based on word pronunciation. A list of 66 common medical terms is organized into 3 columns according to the number of syllables and pronunciation difficulty. The administrator records the number of terms correctly pronounced by the test taker, and the raw count can be converted to 1 of the 4 grade levels: 0 to 3, 4 to 6, 7 to 8, and 9 and above. Criterion validity of REALM is established with Wide Range Achievement Test-Revised (WRAT-R) and other tests in the general domain. Estimate of administration time is under 3 min, making it easy to fit in a busy clinical workflow.

TOFHLA is designed to measure patients’ ability to read and understand what they commonly encounter in the health care setting. It consists of 17 numeracy items and 3 prose passages. The passages are drawn from actual materials a patient may need to read, including instructions for preparation for an upper gastrointestinal series, the patient *Rights and Responsibilities* section of a Medicaid application, and a standard informed consent form. They are converted to a Cloze test with 50 items. Total scores are divided into 3 levels: inadequate, marginal, and adequate. TOFHLA’s correlations with WRAT-R, REALM were tested to establish validity. TOFHLA takes up to 22 min to administer.

Aiming to reduce the administration time, TOFHLA was abridged to an abbreviated version, S-TOFHLA, which takes a maximum of 12 min [12]. A total of 2 passages with 36 items were selected from the full version. S-TOFHLA’s validity is compared with the long version of the TOFHLA and the REALM.

Since the publication of REALM and TOFHLA, many new instruments were derived from them, for different use cases. They were often used as the reference to test for criterion validity. The development process remains largely the same, requiring expert curation and time-consuming validation. For instance, Literacy Assessment for Diabetes [13], Rapid Estimate of Adult Literacy in Vascular Surgery [14], and Arthritis-Adapted REALM [15] were examples in the REALM family. Oral Health Literacy Instrument [16] and Nutritional Literacy Scale [17] followed the design of TOFHLA.

New instruments are constantly developed for particular use scenarios. Examples of specific disease or condition included tests on asthma [18], hypertension [19], diabetes [20], colon cancer [21], and heart failure [22]. Tools for a specific population such as adolescents [23,24] were also developed. In different health domains, Rapid Estimate of Adult Literacy in Dentistry (REALD)-30 [25], REALD-99 [26], Test of Functional Health Literacy in Dentistry [27], Health Literacy in Dentistry (HeLD) [28], and short‐form HeLD-14 [29] targeted dentistry, and Rapid Estimate of Adult Literacy in Genetics [30] measured literacy in genetics.

Another line of research used self-reported comprehension assistance seeking–behavior, as opposed to testing an underlying reading ability, to identify patients with inadequate health literacy. One such study presented 3 questions that can each screen for low literacy [31]. An instrument with a single item was evaluated in a primary care setting to rule out patients with limited health literacy [32].

Among the menagerie of instruments, Medical Term Recognition Test (METER) [33] bears the most similarity to our framework. It included 40 actual medical words and 40 nonwords and required the participant to mark the actual words. This format is generally known as a Yes-No test in the language testing research community. It was proposed in the 1980s as a simple alternative to the traditional multiple-choice method of testing vocabulary knowledge [34]. Scoring of the METER test suffers from a problem that is common to this type of tests: ambiguity in unmarked items. It is not clear whether the participant was uncertain about the item or genuinely did not know it. Our work addressed this problem by explicitly giving various degrees of familiarity with an item as answer options. A second drawback of this tool is that it reused many of the REALM words, rendering the test somewhat redundant.

## Methods

### Study Approval

This study was approved by the Institutional Review Board at the University of Massachusetts Medical School.

### Instrument Framework

We modeled our test framework after the Yes-No vocabulary test. Vocabulary is critical to text comprehension [35]. A meta-analysis showed that vocabulary knowledge most likely played a causal role in comprehension [36]. Another work showed that self-reported comprehension scores improved after lay definitions were provided for medical jargon [37].

In psycholinguistic research, the Yes-No test for vocabulary knowledge usually comprises words at different frequency levels and pseudowords to calibrate for random guessing. Pseudowords are strings of letters that follow the phonotactic and morphological rules of a language but are generally not actual words. The participants are asked to indicate whether they know each of the items.

Although this test format seems simple, creating them is not. Our framework generalized this format by relaxing the need to curate a new set of word and pseudoword items each time a new test is required. Moreover, it can account for uncertainty in the participant’s familiarity with a word. Our framework can also be customized to a particular domain of interest such as dentistry or hypertension.

There are 2 parts to generating a test set under our framework. We start from a vocabulary with their associated occurrence frequencies in a large corpus. The vocabulary is first divided into 10 equally sized tiers based on their frequency. A total of 5 words are then randomly selected from each tier. Next, 2 pseudowords are generated from 2 random words in each tier. The 50 words and 20 pseudowords constitute a complete instantiation of the framework. The options a test taker has for each item are a 4-level Likert scale:

1. I have never seen this word and do not know its meaning.
2. I have seen this word but do not know its meaning.
3. I think I know the word’s meaning, but I am not sure.
4. I am sure I know the word’s meaning.

### Scoring Method

To calculate a score, we measure the agreement between a user and a master. A master perfectly answers all the true words with the most confident value and all pseudowords with the lowest value on the Likert scale. We generalized Cohen kappa (κ) as a measure of agreement, which calculates the observed and chance disagreement:

κ = 1 – q
_o_/q
_e_ (1)

where *q*_*o*_ is the observed disagreement proportion and *q*_*e*_ is the expected disagreement by chance. In an ordinal scale like ours, the proportion can be weighted to account for varying degrees of disagreement [38].

When all the items are considered equal, as in weighted κ, the ratings from the 2 raters can be summarized in a *K* × *K* contingency table, where *K* is the number of categories into which a test item can be assigned. The disagreement proportions can be found from this table by multiplying the different degrees of disagreement *v*_*ij*_, where *v*_*ij*_ is the weight indicating the disagreement when 1 rater assigned *i* whereas the other assigned *j* to an item.

**Figure 1:**
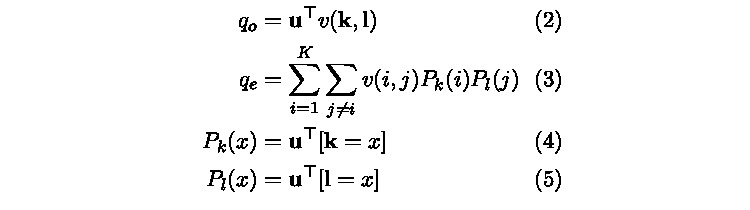
Observed and chance disagreement calculation.

We generalized this agreement by allowing the test items to carry different weights, thus accounting for their prevalence in a corpus and a person’s likelihood of knowing them. We calculate the observed disagreement proportion by summing the individual item’s disagreement, weighted by an item weight. Let u=[*u*_1_, *u*_2_,..., *u*_*N*_] denote the item weights for *N* test items. Note that the weights are normalized such that 0≤ *u*_*i*_≤1 and ∑_*i*__=1_^*N*^*u*_*i*_=1. Let k=[*k*_1_, *k*_2_,..., *k*_*N*_] and l=[*l*_1_, *l*_2_,..., *l*_*N*_] denote the category assignments given to the test items by the 2 raters, respectively. Finally, let *v* (*i*, *j*) denote a function that returns the disagreement weight between categories *i* and *j*. The observed disagreement can be found in equation 2 (Figure 1).

The chance disagreement follows from weighted κ, with the distribution of category assignments for each rater weighted by u as shown in equations 3-5 in Figure 1.

Our generalized κ can be found by substituting the 2 disagreement proportions in equation 1 with equations 2 and 3. The score still has a value range between 0 and 1. An example of the detailed calculations is provided in Multimedia Appendix 1.

### Assessment of Reliability

#### Test Format

In total, 2 parallel instantiations of our framework were created using the same corpus, and scores were calculated using the same disagreement weight and scheme. The 2 tests were shown back to back to participants without demarcations. The test takers were not informed that they were taking 2 equivalent tests.

#### Test Administration

We administered the 2 parallel instantiations of our framework to 100 Amazon Mechanical Turk (AMT) users. They were screened to be from the United States and had an approval rate of at least 90%. We then eliminated answers from users that were not native speakers of English.

Several quality control items were randomly embedded in the test. They were simple and unambiguous questions with only 1 clear and correct answer. They served to identify users that attempted to game our test.

### Assessment of Validity

#### Test Format

We designed a 4-part online questionnaire to validate our health literacy framework. The test consisted of S-TOFHLA, Short Assessment of Health Literacy-English (SAHL-E) [39], our QuikLitE framework, the short form ComprehENotes test [40], and 5 self-reported document difficulty questions.

To generate a set of test items from our framework, we used the Google Books Ngram Corpus [41] as our starting vocabulary. This corpus is a large multilingual collection of digitized books, which were automatically annotated with syntactic information. The English corpus contains approximately 4.5 million volumes and close to half a trillion words. As the earliest volumes date from the 1800s, we selected a subset of books from 2000 and onward to ensure the vocabulary frequencies reflect current language usage patterns. Due to digitization errors, there were non-English words and nonletter symbols in the resulting vocabulary list. We filtered this list to only keep those that appear in WordNet [42]. The required pseudowords were generated by Wuggy [43]. Wuggy’s algorithm operates by building a chain of subsyllabic elements from a large lexicon and then iterates through this chain to search for possible pseudowords. Given a template word, Wuggy can generate pseudowords that match the template’s subsyllabic structure and transition frequency between them.

The ComprehENotes test is an instrument to assess electronic health record (EHR) notes comprehension. It includes 55 snippets of EHR notes from 6 common diseases or conditions and questions generated using the Sentence Verification Technique. In our online setup, we employed the 14-item short-form test.

Texts in the document difficulty questions were randomly selected from Wikipedia articles in the Medicine category. As the writing quality and style vary among Wikipedia articles, we limited our article selection to those that were marked as feature articles. These featured articles, according to Wikipedia editors, are “professional, outstanding, and thorough” and are “a definitive source for encyclopedic information.” Furthermore, only articles designated with top or high importance were considered to eliminate obscure topics. These designations signify “extremely important” or “clearly notable” articles, and there are “strong interests from nonprofessionals around the world” or “many average readers.” Finally, to control for document length, the first few paragraphs of the selected articles were used, and all documents were approximately 300 words long. For each document, the users were asked to rate its difficulty from 1 (easiest to understand) to 10 (most difficult to understand).

Similar to the parallel form reliability test, quality control items that were designed to resemble real test questions were also randomly inserted to filter out cheating test takers.

#### Test Administration

We recruited AMT users to take 3 versions of our online test. The tests differed in the instantiation of our framework and the document difficulty self-assessment. We generated 2 sets of word items from our framework. A total of 2 sets of Wikipedia article excerpts were selected for the document difficulty questions. The 3 versions of the test included different combinations of the vocabulary test and document difficulty test.

A power analysis projected a sample size of 158 to achieve a power of 0.8 with a medium effect size. Published instruments such as S-TOFHLA and SAHL-E, with which we compared in this study, used data from approximately 200 users for validation. We, therefore, recruited 200 users for each of our test version. They were screened in the same fashion as in the reliability assessment.

When scoring our literacy test, we adopted a linear disagreement weight, that is, *v* (*i*, *j*)=| *i* − *j* |. Item weights for true words were based on their transformed frequency in the Google Books Ngram Corpus. Specifically, the word frequencies were converted to a logarithmic scale and standardized. These transformed frequencies were then passed through a logistic function to obtain the item weights. This item weight scheme emphasizes words with high frequencies and applies minimum weight on the rare words. We expect high-frequency words to be known by most native speakers, and unfamiliarity indicates lower language ability and literacy. At the other end of the frequency spectrum, rare words may pose a challenge for most people, holding little power to distinguish the test takers’ vocabulary knowledge. Pseudowords were each assigned a weight equal to the average weight of the true words.

## Results

### Score Distribution

**Figure 2:**
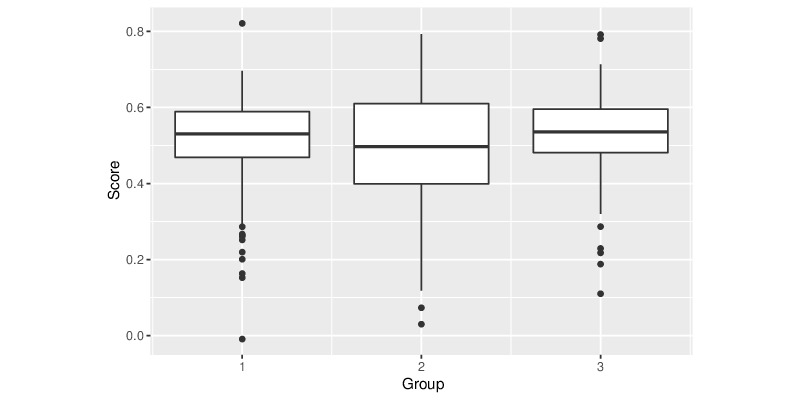
Boxplot of Amazon Mechanical Turk users’ health literacy score according to our framework.

We first present a distribution of health literacy scores as assessed by our framework in Figure 2. Mean scores among users in the 3 groups were 0.514 (SD 0.114), 0.498 (SD 0.154), and 0.528 (SD 0.101).

### Reliability

Of the 100 users that participated in the parallel form test, 90 responses were legitimate. Demographic information of the users is shown in Table 1. The correlation between scores of the 2 equivalent forms was .78 (95% CI 0.69-0.85; *P*<.001), suggesting a high level of reliability.

### Validity

Demographic information of the AMT users is shown in Table 2.

Correlation measured between user score and ComprehenENotes on the 3 groups of users were moderate to decent, shown in Table 3. The correlation coefficients were .61 (95% CI 0.51-0.69), .49 (95% CI 0.38-0.59), and .47 (95% CI 0.35-0.57).

We also measured polyserial correlation between our score and the self-reported document difficulty. The document difficulty scores were reverse coded in the analysis and treated as an ordinal variable. The correlations of the 3 groups were .30 (95% CI 0.17-0.43), .21 (95% CI 0.07-0.34), and .29 (95% CI 0.15-0.41). The weak correlations may be partially explained by the fact that despite given a range of 1 to 10, the AMT users on average rated the document difficulty at 3.8, with an SD of 2.0. As the document excerpts were taken from well-written articles for a wide readership, and over 70% of the users had at least an associate degree, the actual document difficulty ratings concentrated in a narrow range.

Our framework achieved higher correlation with both ComprehENotes and self-reported document difficulty than the 2 existing instruments.

### Subpopulation Differences

We compared the score differences between the subpopulations in our validation data. We divided the data based on gender, race, and age to test differences in the subpopulations. Analysis of variance (ANOVA) showed that there was no significant difference between males and females (*F*_1,79_=2.895; *P*=.09). Older users (>45 years) tended to score higher (*F*_1,579_=21.182; *P*<.001). White users achieved better scores than non-white users (*F*_1,579_=15.462; *P*<.001).

### Ceiling Effect

Existing health literacy instruments may exhibit a ceiling effect, as shown in our data. A total of 52.8% of the users received the full score in SAHL-E and 55.1% in S-TOFHLA, whereas 32.4% scored perfectly in both tests. Furthermore, an overwhelming majority (94.3%) of the users made at most 1 error in either 1 of the tests. This phenomenon was also reported in other studies [44,45]. In contrast, our framework can accommodate a large variation of user health literacy levels. Among different educational attainment levels (high school or less, college, graduate), ANOVA analysis showed that scores under our framework were significantly different (*F*_2,578_=5.605; *P*<.01).

### Administration Time

The median time the AMT users finished our test is reported in Table 4. The majority (90.36%) of users completed the test in less than 5 min. On average, they finished the test 1.5 min faster than S-TOFHLA. Compared with SAHL-E, users took an additional 1.5 min. Among the 30 health literacy instruments with a reported administration time from a catalog [5], our test time is smaller than or equal to 23 measures.

**Table 1:** Demographic information of Amazon Mechanical Turk users in reliability assessment (N=91).

Characteristic		Users, n (%)
**Sex**		
	Female	50 (55)
	Male	41 (45)
**Race**		
	White	74 (81)
	Black	8 (9)
	Hispanic	5 (5)
	Asian	3 (3)
	American Indian/Alaska Native/Native Hawaiian/Other Pacific Islander	1 (1)
**Age (years)**		
	18-24	6 (7)
	25-34	44 (48)
	35-44	24 (26)
	45-54	9 (10)
	55-64	8 (9)
**Education**		
	High school diploma	27 (30)
	Associate	27 (30)
	Bachelor	30 (33)
	Master or higher	7 (8)

**Table 2:** Demographic information of Amazon Mechanical Turk users in validity assessment.

Characteristic		Group 1 (N=192), n (%)	Group 2 (N=196), n (%)	Group 3 (N=193), n (%)
**Sex**				
	Female	89 (46.4)	104 (53.1)	109 (56.5)
	Male	103 (53.7)	92 (46.9)	84 (43.5)
**Race**				
	White	136 (70.8)	154 (78.6)	141 (73.1)
	Black	21 (10.9)	19 (9.7)	20 (10.4)
	Hispanic	12 (6.3)	7 (3.6)	16 (8.3)
	Asian	15 (7.8)	13 (6.6)	14 (7.3)
	American Indian/Alaska Native/Native Hawaiian/Other Pacific Islander	4 (2.1)	1 (0.5)	0 (0)
	Other	4 (2.1)	2 (1)	2 (1)
**Age (years)**				
	18-24	27 (14.1)	22 (11.2)	24 (12.4)
	25-34	83 (43.2)	72 (36.7)	75 (38.9)
	35-44	45 (23.4)	58 (29.6)	58 (30.1)
	45-54	16 (8.3)	26 (13.3)	22 (11.4)
	55-64	9 (4.7)	12 (6.1)	9 (4.7)
	>65	12 (6.3)	6 (3.1)	5 (2.6)
**Education**				
	Less than high school	0 (0)	4 (2)	0 (0)
	High school diploma	53 (27.6)	62 (31.6)	50 (25.9)
	Associate	48 (25)	30 (15.3)	36 (18.7)
	Bachelor	75 (39.1)	74 (37.8)	80 (41.5)
	Master or higher	16 (8.3)	26 (13.3)	27 (14)

**Table 3:** Validity measured by correlation with ComprehENotes and self-reported document difficulty.

Test instrument	ComprehENotes			Document difficulty		
	Group 1	Group 2	Group 3	Group 1	Group 2	Group 3
QuikLitE	.61	.49	.47	.30	.21	.29
SAHL-E^a^	.42	.38	.43	.23	.10	.11
S-TOFHLA^b^	.34	.46	.40	.23	.14	.11

^a^SAHL-E: Short Assessment of Health Literacy-English

^b^S-TOFHLA: Short Test of Functional Health Literacy in Adults.

**Table 4:** Median administration time in seconds.

Test instrument	Group 1	Group 2	Group 3
QuikLitE	173.5	180.5	189
SAHL-E^a^	64	63	64
S-TOFHLA^b^	194.5	199.5	192
ComprehENotes	376	432.5	376

^a^SAHL-E: Short Assessment of Health Literacy-English.

^b^S-TOFHLA: Short Test of Functional Health Literacy in Adults.

## Discussion

### Principal Findings

The responses from AMT users showed high reliability using a parallel form test of our framework. Validity as assessed by correlation with ComprehENotes, an EHR comprehension instrument, was higher than 2 existing health literacy instruments. Our framework also displayed a higher correlation with AMT users’ self-reported document difficulty than S-TOFHLA and SAHL-E. Furthermore, QuikLitE is among the easiest to administer and does not exhibit a ceiling effect.

### Administration

Unlike REALM and its derivatives that rely on word pronunciation checks, our framework can be used in a waiting room without the presence of an administrator or even at home, where the test taker may experience less anxiety. In a clinic, a test can be administered by a nurse with minimal interference to the clinical workflow as it takes less than 5 min. For patients uncomfortable with an electronic device, a paper format can be used, either in a clinic or at home.

Our test can be useful for patients who have seen the material in other instruments. For patients with more exposure to written material, our test can still measure their literacy level. Moreover, if an instrument does not exist for a particular domain of interest, a test can be prepared using our framework.

### Flexibilities

Many aspects of our framework can be easily adjusted to a test designer’s focus. This has several advantages over existing instruments that are static. First, our framework allows for easy instantiation to suit the test designer’s emphasis on a particular subject matter or health care domain. The test may be customized to a particular health care domain or personalized for a specific patient’s need. For example, the education material given to a diabetes patient is different from that given to a cancer patient. Separate vocabularies can be compiled from source texts of various subjects, and subject-specific tests can be created to target patients’ particular needs.

Furthermore, administering the same test over time to monitor a patient’s health literacy can be problematic because repeated testing may result in memorization of the test items, making the measurement unreliable. Creating a unique test on demand with our framework can reduce item repetition, while maintaining similar coverage of word knowledge in vocabulary.

In addition, there is no inherent limit to the number of items that can be included in a test under our framework. The only limit is a test taker and the administrator’s available time. Therefore, to get a broader coverage test of health literacy, a health practitioner can use more words and pseudowords. The same scoring method can be applied without modification.

Finally, our framework can be adapted to other languages. This is especially helpful in languages that REALM style pronunciation tests are difficult to develop because of the phonemic orthographic rules such as in Spanish.

### Limitations

As large corpora are readily available, it is straightforward to create a test set with our framework electronically. However, scoring our test is challenging manually. This may limit its utility when a test is administered in a paper format and a score is needed immediately.

As a test can be generated dynamically, there may be discrepancies with each administration if a new set is created, making comparison difficult. Nevertheless, in our reliability assessment, the median score difference between the 2 equivalent forms is only 0.06. This difference may have little impact on the overall health literacy assessment of a test taker.

In our dataset, the samples were biased toward educated white users. More tests may be needed to assess reliability and validity on underrepresented population in future studies.

Finally, our framework focuses on print literacy. Numeracy and other skills are also recognized as important for managing one’s health. Reading and understanding health-related text is, however, still a critical component to successful engagement with the health care system.

### Conclusions

Over the decades, a plethora of health literacy instruments were published. Designing such instruments are often time-consuming. When a new need arises, such as a new health context or a specific disease or condition, the laborious development process has to be repeated. We, therefore, proposed QuikLitE, a novel framework that can dynamically generate and score a word recognition–based health literacy instrument. Test results with online AMT users showed high parallel form reliability, and it correlated well with ComprehENotes, an EHR comprehension assessment instrument.

## Appendix

Multimedia Appendix 1 An illustrative example of scoring a user’s answer.

## Abbreviations

AMT: Amazon Mechanical Turk
ANOVA: analysis of variance
EHR: electronic health record
HeLD: Health Literacy in Dentistry
METER: Medical Term Recognition Test
REALD: Rapid Estimate of Adult Literacy in Dentistry
REALM: Rapid Estimate of Adult Literacy in Medicine
SAHL-E: Short Assessment of Health Literacy-English.
S-TOFHLA: Short Test of Functional Health Literacy in Adults
TOFHLA: Test of Functional Health Literacy in Adults
WRAT-R: Wide Range Achievement Test-Revised
