# Supplementary material for: QuikLitE, a Framework for Quick Literacy Evaluation in Medicine: Development and Validation
Source: J Med Internet Res. 2019 Feb 22;21(2):e12525. doi: 10.2196/12525 (PMC6406229; doi:10.2196/12525)
Supplement: Multimedia Appendix 1 [file jmir_v21i2e12525_app1.pdf]

1 An Illustrative Example of Scoring a User's Answer

To make the calculation manageable by hand, we assume a test with 5 word items and 5 pseudoword items in this example. Suppose the item weight is $\mathbf{u} = [.15, .14, .09, .08, .04, .1, .1, .1, .1, .1]$.

We encode the four levels of familiarity choice 0 to 3. The master by definition assigns $\mathbf{k} = [3, 3, 3, 3, 3, 0, 0, 0, 0, 0]$ to the test items. Suppose the user's answer is $\mathbf{l} = [3, 3, 2, 2, 0, 0, 1, 0, 0, 0]$.

We start by calculating the observed disagreement, using the linear disagreement weight.

$$\begin{aligned} q_o &= \mathbf{u}^\top |\mathbf{k} - \mathbf{l}| \\ &= .15 \times |3-3| + .14 \times |3-3| + .09 \times |3-2| + .08 \times |3-2| + .04 \times |3-0| + \\ &\quad .1 \times |0-0| + .1 \times |1-0| + .1 \times |0-0| + .1 \times |0-0| + .1 \times |0-0| \\ &= .39 \end{aligned}$$

Next, we calculate the marginal probabilities for the master and the user.

x	0	1	2	3
$P_k(x)$	$.1 + .1 + .1 + .1 + .1 = .5$	0	0	$.15 + .14 + .09 + .08 + .04 = .5$
$P_l(x)$	$.04 + .1 + .1 + .1 + .1 = .44$	.1	$.09 + .08 = .17$	$.15 + .14 = .29$

Then, we can figure out the chance disagreement.

$$\begin{aligned} q_e &= \sum_{i=0}^{3} \sum_{j \neq i} |i-j| P_k(i) P_l(j) \\ &= |0-1| \times .5 \times .1 + |0-2| \times .5 \times .17 + |0-3| \times .5 \times .29 + \\ &\quad |1-0| \times 0 \times .44 + |1-2| \times 0 \times .17 + |1-3| \times 0 \times .29 + \\ &\quad |2-0| \times 0 \times .44 + |2-1| \times 0 \times .1 + |2-3| \times 0 \times .29 + \\ &\quad |3-0| \times .5 \times .44 + |3-1| \times .5 \times .1 + |3-2| \times .5 \times .17 \\ &= 1.5 \end{aligned}$$

Finally, the score of this user is $1 - q_o/q_e = 1 - .39/1.5 = .74$.
